# Unsuspected Fractures

**Published:** 1908-08-01

**Authors:** 


### UNSUSPECTED FRACTURES.

Fractures may elude detection, and, under the *régime* of early passive movements and massage, they often escape the strict confinement that formerly was looked upon as their due; but they ought not to evade suspicion.

Apart from theoretical considerations, the increasing tendency amongst the public to resort to litigation and to prefer charges of malpraxis in cases where there is no question as to the display of "reasonable skill and care" on the part of the practitioner, affords the best of grounds for regarding every accident, however trifling, with suspicion, and for making a mistake on the side of gravity rather than the reverse. It may be said that this is irreconcilable with the spirit of good surgery. It may not be defensible on altruistic grounds, but the responsibility is not with the medical man.

When deformity, preternatural mobility, pain and crepitus are present, there is not much danger of a fracture being overlooked; no one can very well fail to diagnose a transverse fracture of the shaft femur. It may be a very different matter with an impacted fracture of the neck of that bone; there may be no limitation of, or unnatural, movement, no crepitus, no deformity obvious to the uninitiated, and the pain may be—though this is rarely the case—inconsiderable. In one such case the nature of the lesion was disclosed by *x*-rays, only after examination by a surgeon of high repute had failed to discover any adequate explanation of odd pains in the thigh that brought the patient for advice five weeks after the accident. Again, a mother recently brought a girl of eight years to hospital "because she could not put her foot to the ground." She had fallen in the street four weeks before and bent the foot under her; it was swollen and painful, so she was put to bed for a few days, but latterly had been getting about on the sound leg. Physical examination revealed no abnormality beyond a little puffiness about the inner maleolus, and yet on *x*-ray examination an oblique fracture four inches long through the lower end of the tibia was found.

Greenstick fractures and separations of the epiphyses, especially in very young children, may be very difficult to diagnose exactly without radiography, but they do not often escape suspicion. Fractures of the ribs may be overlooked, but probably on the whole they are diagnosed too often rather than too seldom; *x*-rays have taught us this caution, however, that crepitus is not an essential accompaniment of fracture even in these constantly-moving bones, and that a linear crack may cause persistent symptoms. It is particularly the carpal and metacarpal, the tarsal and metatarsal bones that escape suspicion, and for this "that blessed word 'sprain'" has to bear the brunt of the blame. Since January 1, 1906, in the out-patient practice of one surgeon alone, there have been three fractures of the base of the first metacarpal, three of the head of the second metacarpal, one of the head of the fifth, and one of the head of the proximal phalanx of the forefinger, one of the os calcis, and one of the internal cuneiform. All these came to hospital, at various periods after the accident, unsuspected up till that point, in spite of loss of power, restriction of movement, or pain. In only one or two of them was there deformity to suggest fracture, and it was of so slight a degree as to have easily escaped notice.

In the case of the first metacarpal the damage was done by a blow with the clenched fist—a "punch" fracture, in fact. In the case of the os calcis, a man fell twelve feet down a cellar-trap and alighted on his feet; exactly the kind of accident one might expect to produce this particular effect: sometimes the cause is a heavy body dropped upon the foot, sometimes a heavy blow by or upon something held in the hand, by the starting-handle of a motor engine with "back-fire," for example, or a sudden impact upon an iron rod gripped in the hand and supported above the head. The important point to notice is that there is generally some *primâ facie* evidence in favour of a broken bone. Careful inquiry into the precise circumstances and nature of the occurrence, when the patient is intelligent enough to give a reliable account of it, will often serve to prepare the observer's mind for a just appreciation of the true significance, the cumulative force, of signs and symptoms which in themselves and taken individually are of little moment. In the case of the hand it is seldom deformity that excites suspicion, it is the feeble grip or imperfect apposition or flexion. In the foot there is often some deformity, generally referable to a defect of the longitudinal or transverse arches. The most constant feature undoubtedly is persistent pain, which may be elicited by any movement or by some particular muscular action. There may be local tenderness, but its absence certainly does not exclude fracture.

Allusion may be made to fractures of the cervical vertebræ and of the cranial vault. The former are not uncommon; they often give rise to no symptom beyond a stiff neck and perhaps discomfort or pain in swallowing, but it will readily be understood that careful treatment is demanded. In adults there is usually every reason for suspecting fractures of the skull, but it is not always appreciated with what readiness a bone lesion is produced in the thin skulls of children. Neurologists, especially in America, have drawn attention to head injury in childhood as a forerunner of epilepsy.

From the surgical aspect, it is fortunate that the same circumstances which prevent the easy recognition of such fractures as these under review often abrogate the necessity for the strict treatment dictated in the ordinary case, but the modern use of early "movements" must not be allowed to produce the impression that rigid rest is unessential. Passive movement of the neighbouring joints, carried out while the site of fracture is skilfully guarded and the ends of the fragments accurately fixed, is a very different thing from allowing active use of the muscles attached to the broken bone. Rigid rest is the essence of success, and in the case of the hand and foot is best secured by judicious use of removable plaster of Paris or moulded splinting.

